# NRF2 Activation and Downstream Effects: Focus on Parkinson’s Disease and Brain Angiotensin

**DOI:** 10.3390/antiox10111649

**Published:** 2021-10-20

**Authors:** Juan A. Parga, Ana I. Rodriguez-Perez, Maria Garcia-Garrote, Jannette Rodriguez-Pallares, Jose L. Labandeira-Garcia

**Affiliations:** 1 Research Center for Molecular Medicine and Chronic Diseases (CIMUS), IDIS, University of Santiago de Compostela, 15782 Santiago de Compostela, Spain; anai.rodriguez@usc.es (A.I.R.-P.); maria.garcia.garrote@usc.es (M.G.-G.); jannette.rodriguez@usc.es (J.R.-P.); 2 Networking Research Center on Neurodegenerative Diseases (CIBERNED), 28031 Madrid, Spain; 3 Laboratory of Cellular and Molecular Neurobiology of Parkinson’s Disease, CIMUS, Department of Morphological Sciences, University of Santiago de Compostela, R/ San Francisco s/n, 15782 Santiago de Compostela, Spain

**Keywords:** NRF2, antioxidant, heme oxygenase, KLF9, neurodegeneration, Parkinson’s disease, redox signalling, renin–angiotensin system

## Abstract

Reactive oxygen species (ROS) are signalling molecules used to regulate cellular metabolism and homeostasis. However, excessive ROS production causes oxidative stress, one of the main mechanisms associated with the origin and progression of neurodegenerative disorders such as Parkinson’s disease. NRF2 (Nuclear Factor-Erythroid 2 Like 2) is a transcription factor that orchestrates the cellular response to oxidative stress. The regulation of NRF2 signalling has been shown to be a promising strategy to modulate the progression of the neurodegeneration associated to Parkinson’s disease. The NRF2 pathway has been shown to be affected in patients with this disease, and activation of NRF2 has neuroprotective effects in preclinical models, demonstrating the therapeutic potential of this pathway. In this review, we highlight recent advances regarding the regulation of NRF2, including the effect of Angiotensin II as an endogenous signalling molecule able to regulate ROS production and oxidative stress in dopaminergic neurons. The genes regulated and the downstream effects of activation, with special focus on Kruppel Like Factor 9 (KLF9) transcription factor, provide clues about the mechanisms involved in the neurodegenerative process as well as future therapeutic approaches.

## 1. Introduction

Aerobic organisms use oxygen for energy production and product detoxification, typically in organelles such as mitochondria and peroxisomes. As a result of this catabolism, reactive oxygen species (ROS), which are oxygen-derived unstable molecules with a great capacity to react with other molecules, can be generated. In normal conditions, cells can use these ROS to assess the status of many biochemical systems, or even generate ROS in response to certain stimuli as signalling or defence mechanisms. However, excessive ROS production can disrupt normal cell function and structural integrity by reacting with DNA, protein, and lipids. To avoid this damage, cells possess two major endogenous antioxidant systems: enzymatic antioxidants that catalyse ROS detoxification, such as catalase, superoxide dismutase (SOD), glutathione peroxidase, and glutathione reductase, and nonenzymatic antioxidants that donate electrons to reduce ROS, such as glutathione (GSH), uric acid, or vitamins. Oxidative stress is the imbalance between ROS generation and its reduction and it is often the origin or hallmark of many diseases. To reduce oxidative stress, cells evolved different mechanisms such as transcriptional control of antioxidant-related genes, targeted degradation of proteins, and controlled, targeted degradation of the source of ROS by different mechanisms. NRF2 (Nuclear Factor-Erythroid 2 Like 2 or NFE2L2) is known as a master regulator of the antioxidant response [1], as it is known to regulate a great variety of genes with important functions in regulating the oxidative stress response and related pathways.

Neurons are especially vulnerable to oxidative stress. Several neurodegenerative diseases have been shown to have increased levels of oxidative stress [2,3]. Parkinson’s disease (PD) is a neurodegenerative disease characterized by motor symptoms (tremor, bradykinesia, rigidity, postural instability), mainly resulting from the degeneration of dopaminergic neurons in the midbrain nucleus substantia nigra (SN). Although, in most cases, the origin of the disease is unknown, PD has been associated with impairment in many of the neuroprotective mechanism associated with the antioxidant response [4].

In this review, we focus our attention on those mechanisms that increase the production of ROS in neurodegenerative diseases, especially PD, and on how the cells in the nervous system respond to oxidative stress. Recent advances shed light on the regulation of the transcription factor NRF2 in response to ROS, the genes regulated by it, the so-called NRF2 pathway, both in neurons and glial cells, and its implications in PD. Finally, we will address current effort to harness the neuroprotective potential of different approaches involving NRF2 for the treatment of PD.

## 2. Oxidative Stress in the Central Nervous System

Reactive oxygen species (ROS) are generated continually as part of the normal metabolism of the cell, both in the cytoplasm and in different organelles. Mitochondria are the main source of ROS, as molecular oxygen combines with electrons leaking from the mitochondrial respiratory chain, especially complex I [5]. Under physiological conditions, ROS production in mitochondria is relatively low and serves as a signalling mechanism indicative of mitochondrial metabolism [6]. Even “ROS-induced ROS release”, which is a cyclic opening of the mitochondrial permeability transition pore in response to ROS accumulation, is considered a healthy mechanism in cell homeostasis [7]. Peroxisomes also use molecular oxygen as a key reactive component of their normal metabolic functions. Peroxisomes contain several oxidative enzymes that result in hydrogen peroxide production, as well as catalase, an enzyme that, under non-pathological conditions, is able to decompose hydrogen peroxide into water and oxygen [8]. The endoplasmic reticulum is another source of ROS, derived from reactions required for protein folding or from NADPH oxidase (NOX) isoforms found in the endoplasmic reticulum [9]. Other sources of ROS are lysosomes, nuclei, cell membrane and the cytoplasm via NOX, nitric oxide synthases, or as a consequence spontaneous autoxidation [10,11].

Extracellular ROS can be also generated as a signalling mechanism or as a defence system against microorganisms, involving enzymes such as xanthine oxidase, lactoperoxidase, and NOXs [12], the later having also an important function in phagocytic and dendritic cells [13]. NOX activation can increase the production of ROS also indirectly, by activating xanthine oxidase [14] and producing superoxide anion. In general, mitochondria and NOXs are considered the main cellular sources of ROS [15].

Oxidative stress is involved in Parkinson’s disease pathogenesis and progression, involving mitochondria-derived ROS [16], PD-associated genes [17], and neuroinflammation [18]. In the next section, we analyse the possible causes of the vulnerability of the dopaminergic neurons in PD.

## 3. Parkinson’s Disease and NRF2

### 3.1. Dopaminergic Neurons as Vulnerable Targets of Oxidative Stress

Dopaminergic neurons in the SN have several traits that make them especially sensitive to oxidative stress [19,20]: Anatomically, dopaminergic neurons have a long-range neuronal projection with complex dendritic and axonal arborization, coupled with glutamatergic innervation from the subthalamic nucleus, and a high microglia concentration was observed in the SN. Metabolically, dopaminergic neurons are very active, with an autonomous pacemaking activity that requires high oxygen consumption, a finely tuned calcium signalling process, and cellular proteostasis. Dopaminergic neurons in the SN exhibit elevated rates of oxidative phosphorylation in the mitochondria, resulting not only in higher ATP production, but also in ROS generation compared to neighbouring cells in the ventral tegmental area. Additionally, dopamine metabolism, the presence of iron, and low levels of antioxidants make them particularly prone to oxidative stress. Oxidative stress is not only present throughout PD, but has also been detected in early disease stages [21], suggesting an important role in the pathogenesis of the disease.

**Figure 1:**
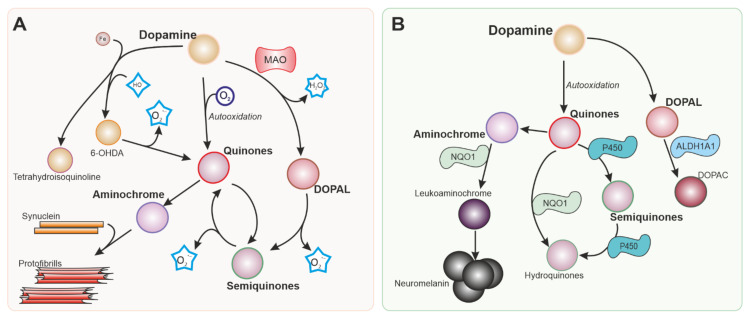
Intracellular dopamine metabolism. Parkinson’s disease is characterized by the degeneration of dopaminergic neurons in the substantia nigra. Dopamine metabolism is responsible in part for the vulnerability of the neurons in this nucleus. Reactions generating reactive oxygen species (ROS) from dopamine and its metabolites are shown in (**A**). Nrf-2 regulates several enzymes represented in (**B**) involved in detoxifying reactions that can mitigate the production of ROS and metabolites that are toxic for dopaminergic neurons. MAO: Monoamine oxidase. 6-OHDA: 6-hydroxydopamine. NQO1: NADPH quinone dehydrogenase 1. ALDH1A: Aldehyde dehydrogenase 1.

Dopamine itself is a source of ROS (Figure 1) that can damage dopaminergic neurons [22]. Dopamine generated in the cytoplasm or uptaken by the dopamine transporter present in dopaminergic neurons is autoxidized, inducing an increase in dopamine quinones that are toxic to the cells. Neurotoxins 6-hydroxydopamine and tetrahydroisoquinoline alkaloids can be produced by a non-enzymatic reaction involving dopamine, H_2_O_2_, and free iron, all of them present in dopaminergic neurons [23].

Monoamine oxidases (MAO) are enzymes that generate H_2_O_2_ as a by-product of the metabolism of catecholamines and indoleamines. While MAO-B is located primarily in glial cells, MAO-A is present in neurons [24], including dopaminergic neurons in the SN, although at relatively low levels [25]. MAO-B is expressed at high levels in astrocytes in the SN, and the H_2_O_2_ produced by this enzyme can cross the cell membrane and affect neighbouring cells, as well as promote excitotoxicity [26].

NOXs are source of ROS, with a special importance in phagocytic cells. Upregulation and release of ROS are hallmarks of activated microglia [27]. Activation of NOX led to an increased microglial activation and dopaminergic cell death in cultures [15]. We demonstrated that NOX activation can increase ROS levels and decrease cell survival also in pure neuronal cultures [28].

### 3.2. Antioxidant Defences and NRF2

Cells have evolved several antioxidant mechanisms to counteract the effect of ROS and avoid damage. Nonenzymatic ROS scavengers include vitamins and their precursors that have direct scavenging effect on ROS and are obtained mostly by food intake. Other molecules with ROS scavenging activity (NADPH, uric acid, glutathione, taurine, thioredoxin…) can, on the contrary, be regenerated by the activity of cellular enzymes.

Enzymes that react to electrophilic chemicals and xenobiotics had been classified as phase I, phase II, and phase III depending on their function. NRF2 is a transcription factor that regulates gene expression of phase I, II, and III enzymes responsible of antioxidant defence [29]. Under normal circumstances, NRF2 is sequestered in the cytoplasm by KEAP1 (Kelch-like ECH-associated protein 1), an inhibitor of its function that facilitates its degradation by ubiquitination [30,31]. In the presence of ROS, reactive nitrogen species, and electrophilic compounds, KEAP1 is modified causing the dissociation of KEAP1 from NRF2, allowing the stabilization of NRF2 and its translocation and accumulation in the nucleus. Alternatively, kinase-mediated phosphorylation of KEAP1 can also induce KEAP1 inactivation and NRF2 translocation to the nucleus [29,32]. Once in the nucleus, NRF2 can bind to the promoter regions of genes containing “antioxidant response element” sequences, promoting the expression of antioxidant genes, including *NRF2* itself [33].

**Figure 2:**
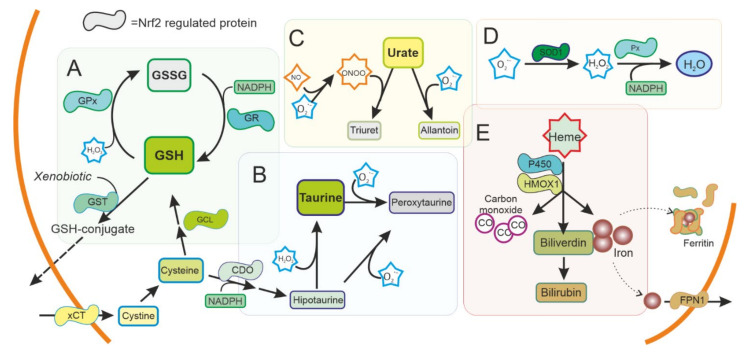
Antioxidant system regulated by NRF2 in the cytoplasm. ROS production is used as a signal molecule and is a byproduct of several reactions in cells that can result in oxidative stress. Cells can use ROS scavengers such as (**A**) glutathione (GSH), (**B**) taurine, (**C**) urate, as well as detoxifying enzymes as (**D**) superoxide dismutase (SOD), peroxidase (Px), (**E**) Heme oxygenase 1 (HMOX1), or cytochrome P450, present in the cytoplasm to reduce ROS and toxic metabolites. NRF2 is regulating and can be regulated by these pathways. GSH: Reduced glutathione. GSSG oxidized glutathione. GST: Glutathione S Transferase. GPx: Glutathione peroxidase. GR: Glutathione reductase. xCT: Cystine-Glutamate exchanger. GCL Glutamate-cysteine ligase. CDO Cysteine dioxygenase. SOD1: Superoxide dismutase type 1. FPN1: Ferroportin.

Dopaminergic neurons are not only exposed to different sources of ROS, but also have compromised antioxidant defence mechanisms. Depending on the source and type of ROS, cells use combinations of ROS scavengers and enzymes that maintain the redox potential of the cell. NRF2 translocates to the nuclei of dopaminergic neurons and increases the transcription of target genes such as Heme Oxygenase 1 (*HMOX1*) and NAD(P)H Quinone dehydrogenase 1 (*NQO1*), which are found in the brains of patients with idiopathic PD [34,35,36]. Besides NRF2 activation in the SN of patients with PD, systemic activation of the NRF2 pathway has been recently reported [37,38,39,40], thus this pathway has been proposed as a marker of PD. In dopaminergic neurons, NRF2 regulates genes that mediate dopamine metabolism (Figure 1B) and various antioxidant systems (Figure 2).

Disorders affecting GSH metabolism are common in major neurodegenerative diseases. In human brains, the levels of GSH peroxidase correlate with the survival of dopaminergic neurons in PD [41] and reduced glutathione levels have been found in the brain of patients with PD [42]. NRF2 regulates the levels of not only GSH peroxidase, but most other key enzymes for GSH synthesis and regeneration (Cystine/glutamate antiporter (xCT), γ-glutamate cysteine ligase (GCL) subunits, glutathione reductase (GR), glutathione S-transferases (GSTs), and others) (Figure 2A). These enzymes have been shown to be affected also in PD brains [43]. Besides GSH peroxidase, there are many other enzymes with peroxidase activity in the thioredoxins superfamily of enzymes that also participate in the control of redox signalling [44]. Most of them are regulated by NRF2, and together function as a signalling system that regulates NRF2 pathway and consequently their own expression [45,46].

Taurine (2-aminoethanesulfonic acid) is the most abundant intracellular amino acid from a very early age in humans, with levels particularly high in excitable tissues that are susceptible to oxidative stress, such as the brain. A reduction in taurine levels has been shown in patients with PD and other neurodegenerative disorders that correlates with the progression of the disease [47]. Taurine can act as an antioxidant and have neuroprotective effects [48,49]. Mechanistically, taurine protects neuronal cells by decreasing superoxide generation from mitochondria, reducing the damage to more sensitive antioxidant systems and, indirectly, by decreasing microglia activation and the oxidative stress associated to microglial NADPH-derived ROS that cause damage in neuronal cells [49,50,51,52]. NRF2 promotes the synthesis of taurine at the expense of cysteine and NADPH, possibly affecting other antioxidant systems, and conversely taurine increases the expression of *NRF2* and downstream genes [53,54].

NADH and NADPH are cofactors essential for maintaining cellular redox homeostasis by providing reducing equivalents to antioxidant enzymes. These molecules are obtained in the tricarboxylic acid cycle in the mitochondria and the pentose phosphate pathway in the cytoplasm. NRF2 is key in regulating the production of these cofactors [55,56], most notably by regulating the pentose phosphate pathway, and tricarboxylic acid cycle intermediates are able to activate the NRF2 pathway [57,58].

Urate is an antioxidant that can scavenge peroxynitrite and hydroxyl radical. Interestingly, urate is the end product of purine metabolism in humans because of the absence of a functional urate oxidase gene. Urate oxidase is present in animal models, and its disruption has been shown to protect dopaminergic cells both in vivo and in vitro [59]. Urate levels are lower in patients with PD [60] and this affects NRF2 expression regulating the antioxidant and inflammatory response [61].

**Figure 3:**
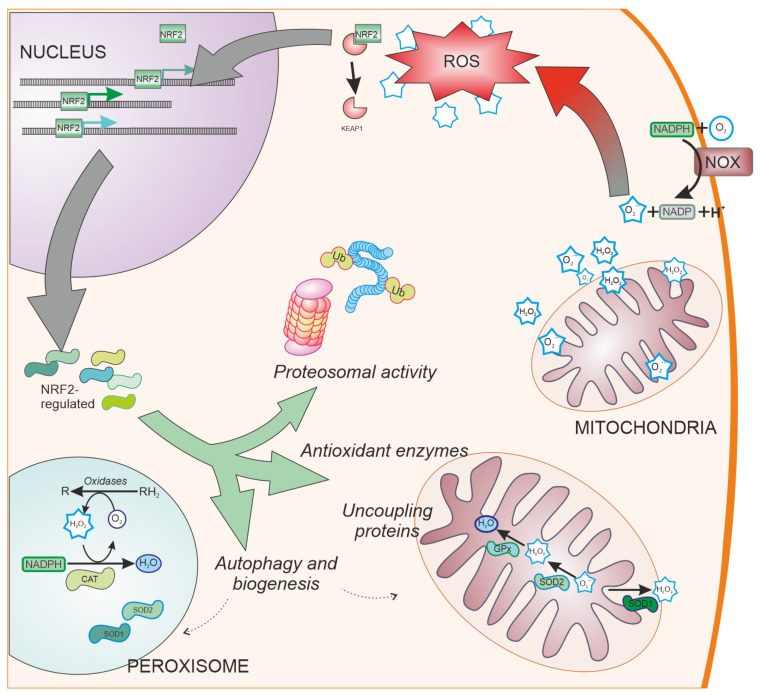
NRF2-regulated systems. Besides ROS scavengers located in the cytoplasm, organelles involved in oxygen metabolism such as mitochondria and peroxisomes, contain NRF2-regulated antioxidant enzymes specialized in the antioxidant metabolism such as catalase (CAT), superoxide dismutase type 1 and type 2 (SOD1 and SOD2, respectively), and peroxidases. Additionally, NRF2 regulates other cell pathways involved in reducing oxidative stress production, such as elimination of proteins by proteosomal degradation or autophagy and organelle biogenesis. NOX: NADPH oxidase. Ub: Ubiquitin. CAT: Catalase.

SOD enzymes are also able to reduce oxidative stress by eliminating superoxide. SOD1, also called CuZnSOD, is located in the cytosol, mitochondrial intermembrane space, and peroxysomes (Figure 3); SOD2 or MnSOD is located mainly in the mitochondria, while SOD3 is mainly extracellular. These enzymes have been shown to be regulated by NRF2 [62], and have been linked to PD [63].

NRF2 is well known to regulate two genes that also have antioxidant activity, *HMOX1* and *NQO1*. The products of these genes metabolize heme and quinone, molecules that can generate ROS, and thus both have been classified as “detoxifying enzymes” [29].

HMOX1 is an inducible enzyme responsible for heme degradation, resulting in carbon monoxide, free iron, and biliverdin (Figure 2E). *HMOX1* expression was upregulated in glial cells in animal models of PD [64] and has been found upregulated in the SN of patients with PD, both in surviving dopaminergic neurons and astrocytes [35]. HMOX1 activity has been associated with cytoprotective effects, although the neuroprotective effect of HMOX1 has been questioned [65,66]. HMOX1 has been shown to have an anti-inflammatory effect and switches macrophages from proinflammatory to anti-inflammatory phenotype [67], suggesting an important role in regulating microglia in PD. Other enzymes related to iron and heme metabolism, including ferritin and ferroportin, are also regulated by NRF2 [68,69]. Levels of ferritin have been found to be decreased in the SN of patients with PD [70]. Ferritin sequesters free iron in microglia with a neuroprotective effect [71].

NQO1 is implicated in the detoxification of quinones (Figure 1B). This is important in dopaminergic neurons since dopamine and other catecholamines can autoxidize to form quinones that can be toxic for dopaminergic neurons before polymerizing to form neuromelanin. 6-hydroxydopamine (6-OHDA), the first dopaminergic neurotoxin discovered, has been also shown to cause dopaminergic neuronal death via quinone formation [72]. Both dopamine and 6-OHDA have been shown to activate the NRF2 pathway and NQO1 [73,74]. NQO1 is localized in dopaminergic neurons in the SN and ventral tegmental area [75]. Elevated NQO1 levels were found in patients with PD [33,76], but NQO1 immunoreactivity is virtually absent when dopaminergic neurons degenerate in advanced stages of the disease [36]. NQO1 has been suggested to protect against several insults associated with PD [77,78]. However, similarly to HMOX1, the neuroprotective effects of NQO1 have been questioned [79].

Another gene of interest for PD is *CYP2D6*. This gene codifies cytochrome P450, a phase I enzyme induced by NRF2 that is highly expressed in liver and brain, where it is involved in drug metabolism. In the brain, it was found expressed at high levels in the SN [80,81], where it is located in dopaminergic neurons [82]. Cytochrome P450 has been shown to have neuroprotective effect in models of PD [83]. Polymorphisms of this enzyme have been associated with PD risk [84] and its expression is decreased in patients with PD compared to age-matched controls [85]. 

Cells have evolved other pathways to reduce oxidative stress by decreasing ROS generation (Figure 3). These pathways include the ubiquitin proteasome system [86], uncoupling mitochondrial proteins [87], and organelle autophagy [88] or biogenesis through PGC-1α [89] and PPARγ [90]. NRF2 is involved in the regulation of these pathways [91,92,93], having shown neuroprotective effects in models of PD [94,95].

### 3.3. Oxidative Stress in Familial Forms of PD: Relationship with NRF2

Oxidative stress is one of the possible mechanisms involved in the pathogenesis and progression of idiopathic forms of PD. Besides those, there are a few genes that have been linked with the disease. For a long time, these genes have been associated with redox imbalance, and their relation with NRF2 is becoming apparent over the more recent years [96].

Synuclein (SNCA) is a major component of the Lewy body, one of the hallmarks of PD, and mutations and even overexpression of the wild type *SNCA* cause familial forms of the disease. Misfolded synuclein causes microglial activation and increased expression of antioxidant response enzymes [97], suggesting a role in regulating NRF2 pathway. NRF2 expression has been shown to be neuroprotective in cellular and animal models expressing α-synuclein [98,99]. In cellular models, downregulation of *NRF2* and *HMOX1* induces synuclein aggregation [100]. Mutant synuclein causes mitochondrial dysfunction and an increase in ROS levels, while NRF2 activation can reduce oxidative stress and ameliorate mitochondrial damage [99].

Mutations in the PARK2 gene parkin (*PRKN*) show impaired ubiquitin protein ligase activity. Cells use parkin to target proteins to be degraded by the UPS and reduce oxidative and endoplasmic reticulum stress [101]. In induced pluripotent stem cells (iPSC)-derived neurons, mutant *PRKN* decreased levels of GSH, increased levels of ROS production, and elevated NRF2 and NQO1. Defects in mitochondria were detected in these neurons, but not in undifferentiated iPSCs nor the fibroblast from patients from which these cells were derived [76].

PINK1 is a protein located in the mitochondrial membrane that can interact with parkin. Together, they regulate mitochondria maintenance by sensing damaged mitochondria. PINK1 has neuroprotective properties by labelling defective mitochondria for selective degradation via autophagy (also called “mitophagy”) [102]. NRF2 can directly upregulate PINK1 in response to oxidative stress [103] and PINK1 reduce mitochondria-derived ROS overproduction by inducing mitophagy. NRF2 activity is upregulated by autophagy, promoting the expression of p62 and PGC-1α, which are key regulators of the recycling of mitochondria and lysosomes [10,104].

*DJ1* gene encodes a highly conserved, ubiquitous protein with functions that are not so well-known. An antioxidant role of DJ1 has been proposed early due to its association with PD [105,106], with prominent expression in glial cells and upregulation of GSH synthesis in PD models [107]. Even before being recognized as a gene responsible for familial forms of PD, DJ1 was found to be oxidized in response to toxins used in PD modelling and microglial activation [108]. DJ-1 directly regulates NRF2 by associating with KEAP1, thus avoiding NRF2 degradation and facilitating its nuclear accumulation [109]. The interaction between DJ-1 and NRF2 is well known and is considered key for the role of DJ-1 in PD pathogenesis [110].

*LRRK2* (Leucine-Rich Repeat Kinase 2) is one of the most prevalent genes associated with familial forms of PD. Wild type *LRRK2* expression increases cell survival in oxidative stress conditions in culture, while viability was decreased in cells carrying a mutant form of *LRRK2* [111]. In individuals carrying the *LRRK2* mutation but without PD, the levels of urate (an NRF2 activator, see above) were higher than in affected patients with the same mutation, suggesting that urate has a protective role and can be used as a biomarker of resistance to PD [112]. An association between NRF2 concentration and UPDRS scores was found in PD carriers of *LRRK2* mutations [113], but there are no statistically significant differences between the levels of NRF2 in CSF of patients with PD with *LRRK2* mutations compared to healthy *LRRK2* carriers.

PARK5 gene, *UCHL-1* (Ubiquitin Carboxyl-terminal Hydrolase L1), is a deubiquitinating enzyme that is affected by oxidative stress in PD [114]. Although association studies between NRF2 and UCHL-1 in PD have not yet been carried out, UCHL-1 is co-regulated with NRF2 in hyperglycaemia models [115], and NRF2 pathway has been proposed as a possible therapeutic approach for traumatic brain injury, where *UCHL-1* is also upregulated [116]; however, a direct link between these two remains to be established. Other genes associated with PD have shown some association with NRF2, but further research is required to stablish NRF2 relevance to other familial forms of PD.

The enzymes and proteins involved in cell protection against oxidative stress, as well as genes associated with familial forms of PD, are, in many cases, present in astrocytes or microglial cells, and not necessarily in neurons: GSH formation requires the interplay between different cells, neuromelanin accumulated inside dopaminergic neurons can be released and activate microglial cells, and strong evidence supports non-cell autonomous degeneration in PD [117,118], including the evidence of cell to cell propagation of synuclein and fibrillary tangles [119]. The role of NRF2 in glial cells is discussed in the next section.

### 3.4. Involvement of Glial Cells in NRF2 Protection

PD affects primarily dopaminergic neurons, but many of the effects of the disease are mediated by glial cells. Many of the neuroprotective effects previously discussed are directly mediated by astrocytes or microglia [120,121,122]. Glial cells typically express higher levels of NRF2 [121,123] and this correlates with higher expression and more variety of antioxidant genes compared with neurons [21,35]. Both astrocytes an microglia express high levels of HMOX1 in early stages of the PD [98], and the role of glial cells has been studied extensively by Cuadrado’s research group in different models of the disease [124,125,126]. In PD, glutamate from the subthalamic nucleus can cause excitotoxicity, but high levels of glutamate also cause the inhibition of the import of cystine from astrocytes, resulting in reduced glutathione levels and a form of cell injury called oxidative glutamate toxicity or oxytosis [127]. Dopamine can activate the NRF2 pathway in astrocytes [128] and activation of the NRF2 in these cells supports the survival of dopaminergic neurons [129,130]. Dopamine also activates NRF2 and promotes iron accumulation in macrophages [131], and efficient iron homeostasis in microglial cells is protective in PD models [132]. Astrocytes can also store iron and reduce iron burden in neurons [133,134], as well as act as glutamate sinks. These mechanisms of iron accumulation are important in ferroptosis, an iron-dependent form of cell death closely related to oxytosis [127,135].

Astrocytic NRF2 is neuroprotective in animal models of PD [136], and the absence of NRF2 in astrocytes could impair the neuroprotection conferred to neurons expressing NRF2 [137]. Given the supporting role that astroglial cells have on neurons, astrocytes are ideal targets to direct therapeutic interventions involving the NRF2 pathway.

The microglial inflammatory response is regulated by different levels of oxidative stress [138]. In microglia, NRF2 pathway induction reduces neuroinflammation and has neuroprotective effects in PD models, being a variety of mechanism involved [139].

## 4. Angiotensin II Signalling

### 4.1. AngII Signaling in the Nervous System

**Figure 4:**
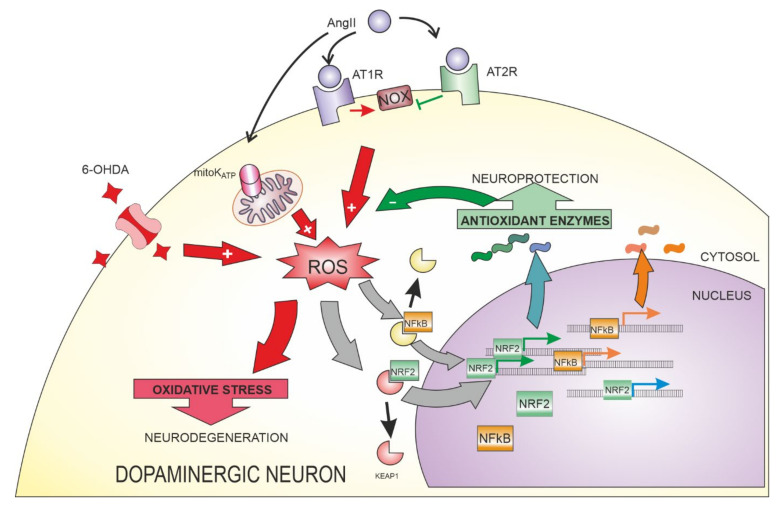
NRF2 activation occurs as a consequences of increased ROS production after treatment with ROS inducers (such as 6-OHDA) and intercellular and intracellular signals (such as Angiotensin II, AngII). AngII can act through different receptors (AT1R, AT2R) and mitochondrial ATP-sensitive potassium channels (mitoK_ATP_) to promote increased ROS production or regulate the oxidative stress by modulating NADPH oxidase (NOX) activity. Cells respond to oxidative stress by regulating different transcription factors: NRF2 signaling is associated with increased antioxidant response, resulting in neuroprotection, while NF-κB gene regulation is often linked to neuroinflammation.

The renin–angiotensin system (RAS), classically associated with systemic blood pressure, has been shown to be important in brain physiology and the pathogenesis and progression of PD. Angiotensinogen is locally synthetized in the nervous system, mainly by astrocytes, and is converted into Angiotensin II (AngII) that acts on microglial cells and neurons. AngII promotes ROS generation [28,140,141] and inflammation [142], mechanisms that are linked to PD origin and progression. AngII signaling is mediated by different receptors in the plasma membrane and intracellularly: Angiotensin type 1 receptors (AT1) mediate pro-oxidative/pro-inflammatory effects (Figure 4), mainly by activating NOX enzymes in the cell membrane and intracellularly [143,144]. Besides NOX activation, AngII can also activate mitochondrial ATP-sensitive potassium channels to increase ROS production from mitochondria [145]. AT1-derived pathways include transactivation of Epidermal growth factor (EGF) receptors, activation of different protein kinases, Phospholipase C (PLC) and NF-κB (Nuclear Factor kappa-light-chain-enhancer of activated B cells) nuclear translocation, among others [146]. Angiotensin type 2 receptors (AT2) receptors (and Mas receptors) have effects opposite to AT1 receptors [147,148], having neuroprotective effects in models of PD [143,149,150], probably mediated by nitric oxide production and NOX inhibition [149,151,152].

### 4.2. Relationship between AngII and NRF2 in Different Tissues

The relationship between AngII signalling and NRF2 has been shown to be quite complicated, with reports indicating that AngII might promote or inhibit NRF2 pathway. NRF2 has been shown to upregulate the expression of angiotensinogen gene and different components of the AngII signalling pathway [153,154], including the expression of NOX in the brain [155]. NOX could act as a double-edged sword, by producing ROS that could increase oxidative stress and damage cells, but also by inducing NRF2 and its neuroprotective pathway, in line with the role of NRF2 as a regulator of the antioxidant defence mechanism: If NOX are the main effectors of AngII signalling, the subsequent increase in ROS would induce NRF2 translocation and the activation of its genetic program. However, Ang(1-7) can also activate the NRF2 pathway [156] and upregulate its targets catalase and SOD [157].

Excessive AngII can have deleterious effects on the cardiovascular system. AngII could be harmful to cardiomyocytes, and this damage can be aggravated in the absence of NRF2 [158]. Angiotensin-induced damage was prevented by activation of the NRF2 pathway in cardiomyocytes [159,160], endothelial cells [161,162], and smooth muscle cells [163,164,165]. Similar results were reported in the liver [166], testicles [167], and lung [168].

AngII effects on kidneys are, together with the effect at the cardiovascular level, probably the best well known targets of the classical RAS system effects. Angiotensin has damaging effect in diabetic kidney disease and chronic kidney disease [169,170], and NRF2 activation has been proposed as a possible therapy, but with controversial results. Contrary to what could be expected, in some models of these diseases, nuclear translocation of NRF2 and expression of target genes are reduced [170] or linked to damaging effects [171]. Surprisingly, AngII has been shown to reduce *NRF2* expression in models of renal disease [172,173] and hypertension [174,175], suggesting a defect in the NRF2 antioxidant system. A possible role of glucose has been proposed to explain these paradoxical results: in OLETF (Otsuka Long-Evans Tokushima Fatty) rats, a model of hypertension, high glucose levels might impair NRF2 pathway [176], and blocking AT1 might actually upregulate *NRF2* and improve mitochondrial function, having antioxidant effects. Insulin is able to reduce hypertension and oxidative stress, and at the same time, to inhibit angiotensinogen expression and the NRF2 pathway [177]. In Akita mice, a model of diabetes with associated hyperglycaemia and hypertension, overexpression of *NRF2* reduced the protective arm of the renin–angiotensin system by decreasing Angiotensin-converting enzyme 2 (ACE2), Angiotensin 1–7, and Mas receptor expression. NRF2 inhibition resulted in upregulation of this neuroprotective arm of the AngII system, and downregulation of angiotensinogen and ACE2 [178].

As mentioned at the beginning of this section, AngII in the nervous system has a damaging effect through AT1 receptor activation. AngII increases the toxic effect of 6-OHDA in animal models of the disease [148], which is blocked by antagonists of AT1 receptors. This toxicity is evidenced by the levels of lipid peroxidation and protein oxidation, suggesting that oxidative stress is involved in the observed effects. We observed an increase in oxidative stress affecting cells in culture in response to AngII or 6-OHDA, using different ROS sensitive probes. Combination of 6-OHDA and AngII again produced the maximal effect, exceeding the levels induced by any of the treatments alone [28]. AngII triggers an antioxidant hormetic adaptation in dopaminergic neurons [143,144]. These effects have been shown to be mediated by PGC-1α, SIRT1, and IGF1. These three effectors can regulate NRF2, usually via the GSK3 pathway, and NRF2 can also regulate PGC-1α and SIRT1 in a feedback loop. Moreover, SIRT1, PGC-1α, and PPARγ are involved in HMOX1 regulation [179]. We also found that AngII and 6-OHDA induce an increase in the levels of ROS, and this increase was associated with an upregulation in NRF2-regulated genes HMOX1 and NQO1 in dopaminergic neurons, both in culture and in vivo [28]. In mice, activation of *Nrf2* attenuates the damaging effects of AngII [180]. NRF2 has a direct protective effect by increasing the levels of antioxidant proteins. Moreover, NRF2 can decrease the expression of proteins involved in the AngII-AT1-NOX pathway [181] or improve mitochondrial function, and these could lead to a reduction in ROS generation. AngII-induced effects could be dependent on the activated cell type, as AngII stimulation downregulated NRF2 and HO-1 expression in astrocytes [182]. Controversial results were obtained in the Neuro2A cell line, where AngII and ACE2 independently induced NRF2 translocation to the nuclei, but no synergistic effect was observed [180]; in a different report, AngII reduced the expression of *NRF2* mRNA that is reversed by an AT1 antagonist, but the link remains unclear [183].

Additional support for a direct relationship between NRF2 and AngII comes from the *Nrf2* knockout rat (Nrf2^(−/−)^). This animal has a deletion in the *Nrf2* gene that results in a reduction in the expression of neuroprotective genes such as HMOX1, catalase, or SOD1 and SOD2. The lack of the antioxidant defence system caused an endothelial dysfunction and salt induced oxidant stress in normotensive rats. The results of this work suggest that NRF2 is mediating the “paradoxical protective effect of low-dose angiotensin II infusion” [184].

There is a co-regulation between the neuroprotective signalling associated with NRF2 pathway and the NF-κB pathway [185]. NRF2 and NF-κB behave as antagonistic transcription factors, (i.e., NRF2 can inactivate NF-κB signalling [186] and NF-κB antagonizes NRF2 by depriving it from required co-transcription factors and epigenetically silencing its target genes [187]) and have combined but opposite effects on the survival of dopaminergic neurons [188,189]. This can provide a causal link between oxidative stress and neuroinflammation. AngII (and other oxidative stress inducers) could activate not only the neuroprotective NRF2 pathway, but also NF-κB pathway.

## 5. Role of NRF2-Induced KLF9 Expression

The nuclear NRF2 observed in surviving neurons has been interpreted as the ROS-induced cellular response aimed to improve dopaminergic survival [33,34]. However, NRF2-mediated effects might not be sufficient to protect all nigral neurons. In conditions of excessive oxidative stress, despite the antioxidant mechanisms elicited by the NRF2-mediated gene expression, ROS levels could still be too high and cause cell damage or even death. NRF2 can also regulate many other genes with a wide variety of functions. For many of these, their role in the progression of PD is still unknown.

**Figure 5:**
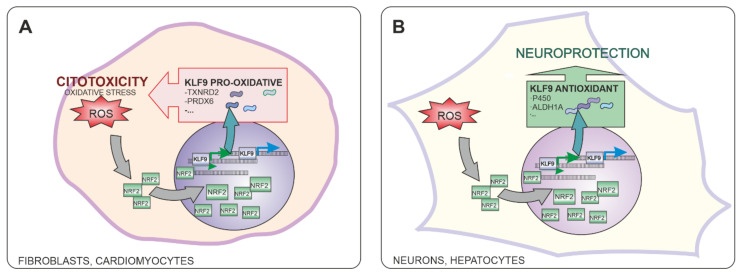
Kruppel-Like Factor 9 (KLF9) transcription factor is expressed as a consequence of excessive NRF2 activation. KLF9 has been shown to have different effects in different cell types: While in some cells, KLF9-mediated gene expression results in increased ROS production and cytotoxicity (**A**), KLF9 has been shown to reduce oxidative stress and promote neuroprotection in neurons and hepatocytes (**B**).

*KLF9* (Kruppel Like Factor 9) is a transcription factor that can be induced by different stressors and by an excessive activation of the NRF2 pathway [190]. *KLF9* is highly expressed in brain and is necessary for the development and survival of Purkinje neurons [191]. It also has beneficial effects on the proliferation of iPSC-derived neurons [192] and differentiation and maturation of neuronal cells [193,194]. KLF9 regulates the expression of genes involved in neuroprotection, neuroregeneration, and neurogenesis in animal models of brain damage [194,195], mediating some of the neuroprotective actions of thyroid hormones in the brain. Results obtained in our laboratory also support the hypothesis that KLF9-mediated gene regulation reduces oxidative stress and promotes neuronal survival: we observed a reduction in the levels of superoxide and increased viability in cell lines expressing KLF9 compared to controls [28]. However, previous studies have reported that KLF9 can increase ROS generation, causing oxidative stress, tissue injury, and cell death. Several other studies have also observed that KLF9 has deleterious effects on the survival of non-neuronal cell types [190,196,197]. In macrophages, KFL9 can induce apoptosis and decrease in prostaglandin production, but also promote the survival of co-cultured cells [198]. This suggests that gene regulation mediated by the transcription factor KLF9 may be dependent on the epigenetic program of the cell expressing it (Figure 5).

Cytochrome P450, a phase I enzyme regulated by NRF2, could be key to explain these results. KLF9 expression (induced by NRF2) has a beneficial effect in hepatocyte survival [199]. Hepatocyte expression of *CYP2D6* (the gene that codes cytochrome P450) is regulated indirectly by NRF2 [200,201] as NRF2 induces KLF9, and KLF9 upregulates cytochrome P450 expression [202,203]. Cytochrome P450 is not only present in the liver, but also in the brain and in dopaminergic neurons, and has been associated with PD risk, as indicated above. The results in hepatocytes suggest that cytochrome P450 may be responsible for the reduced oxidative stress and neuroprotection in dopaminergic cells and other neurons, but further research should be conducted to support this hypothesis.

Examples of transcriptional regulation by KLF9 in other tissues include genes that are key for proliferation, differentiation, and survival in dopaminergic neurons, such as *BDNF* (Brain derived neurotrophic factor), *ALDH1A1* (Aldehyde Dehydrogenase 1, which mediates the detoxification of the dopamine intermediate DOPAL and synthesis of retinoic acid), *RXRA* (Retinoid X receptor alpha, which mediates retinoic acid signalling), and *PTCH1* (Patched 1, Hedgehog pathway receptor that has effects on cell proliferation and apoptosis in PD models) [204]. However, differences are expected even between neuronal types [205] and no gene expression studies have been done so far in dopaminergic neurons.

## 6. Therapeutic Approaches Using NRF2 for Neuroprotection

NRF2 is a master regulator of genes involved in ROS degradation and detoxification. Given its importance in PD and other neurodegenerative disease, NRF2 is an ideal target candidate for the treatment of neurodegenerative diseases and the targeted discovery of new drugs [185,206], although some cautions should be considered as NRF2 activation has also been linked to the survival of some carcinogenic cells.

Many compounds with known neuroprotective effects have shown to exert their actions through the activation of the NRF2 pathway (Table 1) [207,208,209] and chemical screening used to discover new cytoprotective drugs show that their effect is mediated by the NRF2 pathway. Some screening assays have been designed to find NRF2 activators [128,210] or modifying known NRF2 activators, such as sulforaphane [211], flavonoids [212], or chalcones [213], among others, to provide therapeutic effects in cellular models of PD [214,215]. In other cases, the involvement of NRF2 pathway was discovered after finding that the compound had an effect on microglial neurotoxicity [216], highlighting its importance in different neurodegenerative diseases. The effects of some of these assays were also translated into animal models of PD showing neuroprotective results [125,217,218] and supporting this approach in a pre-clinical setting.

The NRF2 pathway could regulate not only oxidative stress, but also neurogenesis. Most of the neurons in the nervous system are generated before birth, but there are a few niches where neural stem cells are still present in adult individuals. Since the neurodegeneration of dopaminergic cell is responsible for many of the symptoms of PD, the regulation of neurogenesis is another therapeutic target for treating this disease. Upregulation of NRF2 has been shown to maintain the proliferative capacity of neural stem cells [238,239]. This effect is reminiscent of the role of NRF2 in cancer cells, where it promotes cell division [240]. AngII has been suggested to promote the senescence [156,241] or proliferation [242] of stem cells, depending on the cell type, receptor subtype, or the signalling evoked. We have recently demonstrated that AT2 activation induces neurogenesis in the adult rodent subventricular zone [243].

Based on their origin, most NRF2 inducers that are being tested for neuroprotection of dopaminergic cells can be classified as exogenous (such as phytochemicals or pharmaceutical drugs) [29,45]. However, endogenous molecules that regulate NRF2 signalling could also be harnessed for neuroprotective therapies. AngII, nitric oxide, prostaglandins, and products of lipid metabolism (such as 4-hydroxynonenal and nitro-fatty acids) affect the activity of the NRF2 pathway. Therapies directed towards these compounds would indirectly regulate NRF2 pathway.

Besides chemical drugs, therapies such as physical exercise have shown protective effects on PD. We have demonstrated that exercise has beneficial effects in PD models [244,245], and some of the beneficial effects could be mediated by NRF2, as this is a known target of physical exercise in models of PD and patients [233,234,235,236,237].

## 7. Conclusions

NRF2 expression regulates the expression of a great number of genes, mainly associated with an antioxidant response. This suggests that this pathway is interesting for the treatment of many diseases, including neurodegenerative diseases. The relationship with PD is well stablished, and pre-clinical models suggest that it is an interesting target for the discovery of new drugs. However, the relationship between the NRF2 pathway and other signalling mechanisms is not so well understood. The role of AngII in the activation of the NRF2 pathway seems to be determined by the cell type or model used, with confounding effects of different pathologies in the AngII initiated signalling pathways. Similar to this, KLF9 effect on oxidative stress seems to be linked to the cell types and their epigenetic status. Future approaches should consider these observations and take into account the complex regulation of AngII and NRF2 in the context of neurodegenerative diseases and aging.

## Figures and Tables

**Table 1:** NRF2 modifiers assayed for PD therapy.

Type of Approach	Compound	References
Exogenous chemicals	Sulforofane	[211,219,220]
	Flavonoids	[212,221]
	Polyphenols	[137,222]
	Chalcones	[213,223]
Endogenous signaling	Nitric oxide	[224,225]
	Prostaglandins	[226,227]
	Lipid metabolites	[228,229]
	RNA	[230,231,232]
Alternative approaches	Physical exercise	[233,234,235,236,237]
